# History of fatigue in multiple sclerosis is associated with grey matter atrophy

**DOI:** 10.1038/s41598-019-51110-2

**Published:** 2019-10-14

**Authors:** Miklos Palotai, Aria Nazeri, Michele Cavallari, Brian C. Healy, Bonnie Glanz, Stefan M. Gold, Howard L. Weiner, Tanuja Chitnis, Charles R. G. Guttmann

**Affiliations:** 1 Center for Neurological Imaging, Department of Radiology, Brigham and Women’s Hospital, Harvard Medical School, Boston, Massachusetts USA; 2 Mallinckrodt Institute of Radiology, Washington University School of Medicine, St. Louis, Missouri USA; 3 Partners Multiple Sclerosis Center, Department of Neurology, Brigham and Women’s Hospital, Harvard Medical School, Boston, MA USA; 4 Biostatistics Center, Massachusetts General Hospital, Harvard Medical School, Boston, Massachusetts USA; 5 Charité Universitätsmedizin Berlin, Klinik für Psychiatrie und Medizinische Klinik m.S. Psychosomatik, Berlin, Germany; 6 Institut für Neuroimmunologie und Multiple Sklerose (INIMS), Universitätsklinikum Hamburg-Eppendorf, Hamburg, Germany

**Keywords:** Multiple sclerosis, Neurodegeneration

## Abstract

Fatigue in multiple sclerosis (MS) has been associated with brain damage with low replicability. Temporal fatigue fluctuations have not been considered. We assessed whether sustained fatigue (SF) associates more strongly with grey matter (GM) changes than reversible fatigue (RF). Patients were stratified into three groups according to historical fatigue levels: SF (n = 30, i.e. patients who reported fatigue at the latest ≥2 assessments), RF (n = 31, i.e. patients not fatigued at the latest assessment, but reported fatigue previously), and never fatigued (NF, n = 37). Groups were compared for brain GM volume using cross-sectional voxel-based and volumetric analyses of 3T T1-weighted MRI. Confounding effects of depression and related medications were also investigated. SF and RF patients showed similar anatomical distribution of GM atrophy. While we robustly replicated the anatomical patterns of GM atrophy described in previous work, we also found an association between hippocampal atrophy and fatigue. Depression showed confounding effects in frontal, parietal, occipital, accumbal and thalamic regions. Assessed treatments showed confounding effects in frontal, parietal and striatal areas. Our results suggest that history of clinically-relevant fatigue in currently non-fatigued patients is associated with GM atrophy, potentially explaining inconsistent findings of previous studies that stratified patients using a single fatigue assessment.

## Introduction

Fatigue is among the most disabling symptoms in multiple sclerosis (MS)^1^, and is associated with disease progression^2^. Neural, immune, endocrine and metabolic mechanisms have all been proposed to play a role in the development of fatigue^1^. Neuroimaging studies have associated fatigue with brain damage in MS patients^1,3–13^, but the anatomical patterns were not consistent between studies, and a few studies could not show a structural association, at all^14–18^.

A previous study suggested that fatigue is highly variable over time: 54% of MS patients fluctuated between “fatigued” or “non-fatigued” states, 27% were persistently “fatigued” and 19% were persistently “non-fatigued” over a course of 2 years, during which fatigue was assessed every 6 months^19^. Therefore, a single assessment might not be sufficiently representative and robust to categorize a patient into a “fatigued” or “non-fatigued” group. A limitation of previous MRI studies has been the lack of accounting for fluctuations of fatigue over time, which may explain discrepancies between their results.

We hypothesize that the pathogenesis of persistent fatigue differs from that of fluctuating fatigue: Persistent fatigue over years is more likely to be caused by irreversible neurodegeneration, whereas fluctuating fatigue may reflect reversible pathobiological changes (e.g. inflammatory cytokine and hormone levels). We therefore defined the following three patient groups considering longitudinal fatigue assessments over the course of up to 14 years: never fatigued (NF), sustained fatigue (SF) over the most recent two years, and reversible fatigue (RF) (presently not reporting fatigue, but did in the past). We anticipated that SF patients would show more pronounced gray matter (GM) damage than RF and NF patients. Since depression is a common comorbidity^1^, we also investigated the effects of depression and medications, that may influence the perceived level of fatigue and/or depression, on the relationship between fatigue and GM damage.

## Results

There was no significant difference between the SF, RF and NF groups in age, sex, disease duration, EDSS, and time between MFIS assessment and MRI scan (Table 1). At the most recent measurement, SF patients showed significantly higher total and subscale MFIS scores (p < 0.001 vs RF, p < 0.001 vs NF); total CES-D score (p < 0.001 vs RF, p < 0.001 vs NF); as well as CES-D subscale scores, i.e. somatic symptoms (p < 0.001 vs RF, p < 0.001 vs NF), depressed affect (p = 0.032 vs RF, p < 0.001 vs NF), anhedonia (p = 0.020 vs RF, p < 0.001 vs NF) and interpersonal concerns score (p = 0.020 vs RF, p < 0.001 vs NF) compared to the other two groups (Table 1). 20 out of the 98 patients (14 SF, 5 RF, 1 NF) had clinically significant (CES-D ≥16) depression. These variables were not significantly different between RF and NF, but there was a trend showing higher scores in RF patients (Table 1).

**Table 1:** Comparison of demographic and clinical variables of the CLIMB cohort as well as MS patients with sustained, reversible or no fatigue selected from the CLIMB cohort.

	CLIMB (n = 2421)^a^	SF + RF + NF (n = 98)^b^	SF (n = 30)	RF (n = 31)	NF (n = 37)
Age (years)	49.5 (12.3)	49.3 (8.4)	48.9 (8.9)	49.5 (9.8)	49.4 (6.7)
Gender (female/male) (%)	73/27	78/22*	80/20	74/26	78/22
Disease duration (years)	13.7 (8.5)	17.2 (7.5)**	17.7 (6.6)	16.9 (8.0)	17.0 (8.1)
Disease category (RRMS/SPMS/PRMS/other) (%)	71/17/1/11	87/13/1/0	87/13/0	84/13/3	89/11/0
Time between MFIS assessments and MRI scan (months)	N/A	3.9 (5.2)	2.9 (4.6)	3.6 (5.4)	5.0 (5.4)
EDSS	2.6 (2.3)	2.0 (1.5)	2.3 (1.5)	2.0 (1.8)	1.8 (1.0)
MFIS-total^c^	26.4 (17.9)	28.9 (17.5)	50.3 (7.6)***	23.2 (9.8)	16.2 (10.6)
MFIS-cognitive subscale^c^	11.9 (8.5)	13.9 (9.0)**	24.1 (5.5)***	11.0 (5.9)	8.0 (5.9)
MFIS-physical subscale^c^	12.3 (9.0)	13.0 (8.4)	22.3 (4.4)***	10.7 (6.0)	7.4 (6.0)
MFIS-psychosocial subscale^c^	2.2 (2.0)	2.1 (1.8)	3.9 (1.7)***	1.5 (1.2)	1.1 (1.2)
CES-D-total [number of patients with CES-D >16]^c^	10.3 (8.8) [177]	9.9 (7.9) [20]	15.9 (8.7)*** [14]	8.8 (7.0) [5]	5.8 (4.2) [1]
CES-D-somatic symptoms subscale^c^	N/A	3.9 (3.0)	6.7 (2.9)***	3 (2.2)	2.4 (1.8)
CES-D-depressed affect subscale^c^	N/A	1.5 (2.0)	2.6 (2.6)***	1.4 (1.7)	0.7 (1.3)
CES-D-anhedonia subscale^c^	N/A	3.2 (2.7)	4.9 (2.8)***	3.1 (2.7)	2.0 (2.0)
CES-D-interpersonal concerns subscale^c^	N/A	0.3 (0.7)	0.7 (0.9)***	0.2 (0.7)	0.1 (0.4)
Brain white matter lesion load (cm^3^)	N/A	6.7 (8.6)	9.7 (11.8)***	6.9 (8.5)	3.9 (3.7)

Results are presented as mean (standard deviation).

Only the most recent MFIS scores are presented in the table and were used in the statistical analyses.

*p < 0.05 versus CLIMB using Pearson’s chi-square test.

**p < 0.05 versus CLIMB using Wilcoxon rank sum test.

***p < 0.05 versus RF and NF using one-way ANOVA.

^a^To calculate the demographic data of the CLIMB cohort, the database was queried on 12/07/17.

^b^To select SF, RF and NF patients for MRI analysis, the CLIMB database was queried on 02/19/16.

^c^MFIS and CES-D were measured only in the Quality of Life (QOL) subset of the CLIMB cohort.

Abbreviations: CLIMB: Comprehensive Longitudinal Investigations of MS at the Brigham and Women’s Hospital, SF: patients with sustained fatigue, RF: patients with reversible fatigue, NF: never fatigued patients, RRMS: relapsing-remitting MS, SPMS: secondary progressive MS, PRMS: progressive relapsing MS, EDSS: Expanded Disability Status Scale, MFIS: Modified Fatigue Impact Scale, MFIS-cog: cognitive subscale score of MFIS, MFIS-phys: physical subscale score of MFIS, MFIS-psych: psychosocial subscale score of MFIS, CES-D: Center for Epidemiologic Studies Depression Scale, ANOVA: analysis of variance.

Disease duration (p < 0.0001) and female to male ratio (p = 0.005) were significantly higher in the pooled SF + RF + NF cohort comparted to the CLIMB cohort, while no significant difference was present for age and EDSS (Table 1). The selected dataset was well matched to the QOL subset of the CLIMB for total score and physical and psychosocial subscale scores of MFIS, but had higher cognitive subscale scores (p = 0.035) (Table 1).

In the pooled SF + RF + NF cohort, total MFIS showed significant correlation with CES-D (p < 0.0001, rho = 0.51) and EDSS (p = 0.044, rho = 0.20), but CES-D and EDSS were not significantly inter-correlated (p = 0.64, rho = −0.05). In the QOL subset, total MFIS score was significantly correlated with CES-D (p < 0.0001, rho = 0.90) and with EDSS (p = 0.0001, rho = 0.14), but CES-D and EDSS (p < 0.0001, rho = 0.16) also showed significant, albeit weak correlation.

Total brain WMLL was significantly higher in SF versus RF and NF patients (p = 0.018), but there was no difference between RF and NF patients (Table 1).

**Figure 1:**
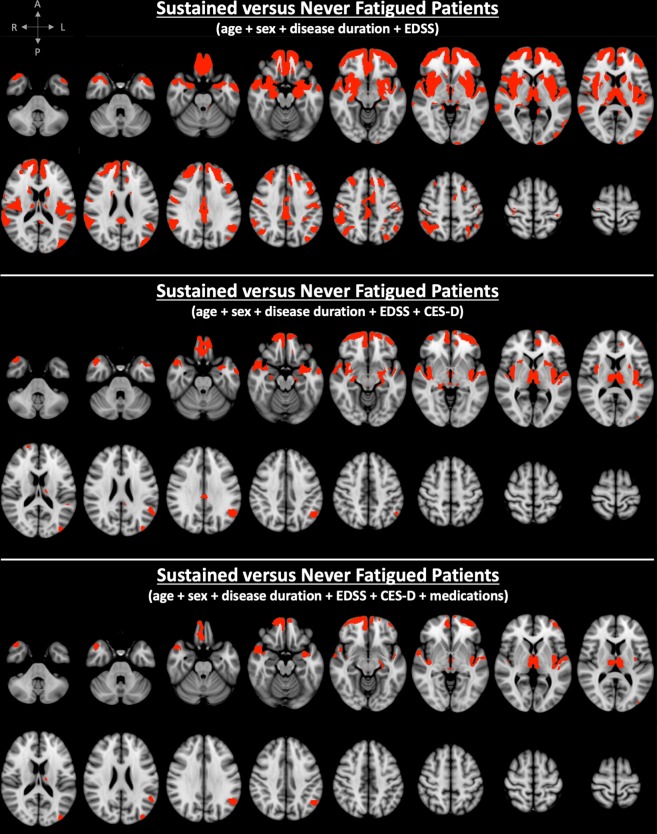
Spatial distribution of clusters with significant atrophy overlaid on the ICBM 152 template in MS patients with sustained fatigue (SF) compared to never fatigued (NF) MS patients. Correction was made for age, sex, disease duration and Expanded Disability Status Scale (EDSS) score (top), and for Center for Epidemiological Studies - Depression score (CES-D) (middle), as well as for medication (bottom). (red labels = family-wise error + Bonferroni-corrected p value < 0.017).

**Figure 2:**
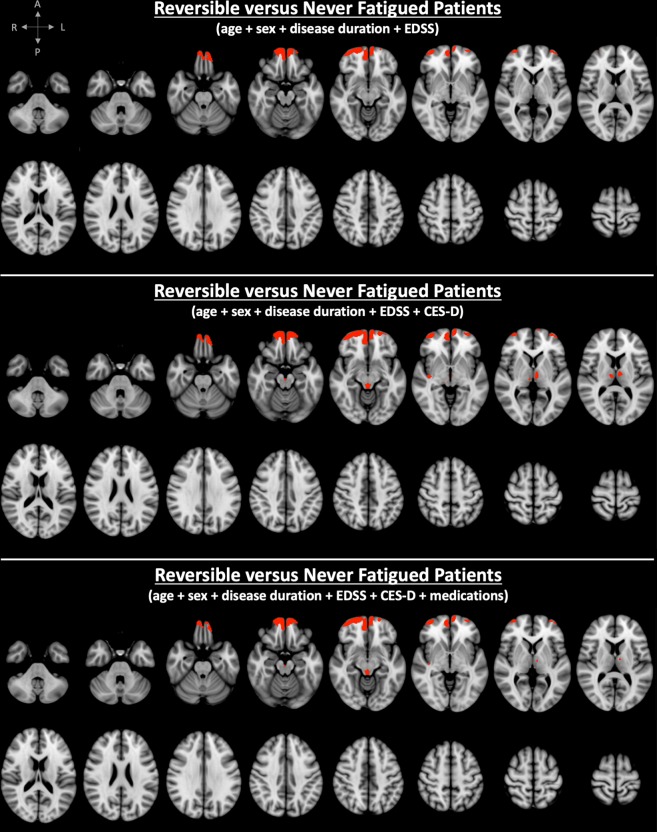
Spatial distribution of clusters with significant atrophy overlaid on the ICBM 152 template in MS patients with reversible fatigue (RF) compared to never fatigued (NF) MS patients. Correction was made for age, sex, disease duration and Expanded Disability Status Scale (EDSS) score (top), and for Center for Epidemiological Studies - Depression score (CES-D) (bottom), as well as for medication (bottom) (red labels = family-wise error + Bonferroni-corrected p value < 0.017).

VBM analysis adjusted for age, sex, disease duration, and EDSS showed significantly lower volumes in several cortical regions encompassing all four brain lobes and the insula, along with subcortical structures (caudate, putamen, thalamus, amygdala and hippocampus) on both sides in SF compared to NF patients (Fig. 1 and Table 2). Comparison between RF and NF patients showed signal (ie, lower GM volume) only in bilateral frontal cortical areas (Fig. 2 and Table 2). We found no significant differences between SF and RF patients. SF patients showed atrophy in 33 GM areas (29 bilateral), whereas RF patients showed atrophy in 4 GM areas (4 bilateral) (Table 2). The total number of significantly different GM voxels was 20-times larger in the SF versus NF contrast compared to the RF versus NF contrast (Table 2).

**Table 2:** Brain GM areas with significant volume loss in SF versus NF patients as well as in RF versus NF patients when controlling for age, sex, disease duration, EDSS ± CESD ± medication (FWE + Bonferroni-corrected p < 0.017).

	Adjusting for age + sex + disease duration + EDSS		Adjusting for age + sex + disease duration + EDSS + CESD		Adjusting for age + sex + disease duration + EDSS + CESD + medication	
	SF vs NF	RF vs NF	SF vs NF	RF vs NF	SF vs NF	RF vs NF
Frontal pole	bilateral	bilateral	bilateral	bilateral	bilateral	bilateral
Superior frontal gyrus	bilateral	N/A	N/A	N/A	N/A	N/A
Middle frontal gyrus	bilateral	N/A	N/A	N/A	N/A	N/A
Inferior frontal gyrus	bilateral	N/A	N/A	N/A	N/A	N/A
Frontal-orbital cortex	bilateral	bilateral	bilateral	bilateral	bilateral	bilateral
Frontal-medial cortex	bilateral	bilateral	bilateral	bilateral	bilateral	bilateral
Cingulate gyrus	bilateral	N/A	bilateral	N/A	N/A	N/A
Paracingulate gyrus	bilateral	bilateral	bilateral	bilateral	bilateral	bilateral
Precentral gyrus	bilateral	N/A	N/A	N/A	N/A	N/A
Postcentral gyrus	bilateral	N/A	N/A	N/A	N/A	N/A
Insula	bilateral	N/A	bilateral	right	bilateral	right
Temporal pole	bilateral	N/A	bilateral	N/A	bilateral	N/A
Superior temporal gyrus	bilateral	N/A	bilateral	N/A	bilateral	N/A
Middle temporal gyrus	bilateral	N/A	bilateral	N/A	bilateral	N/A
Inferior temporal gyrus	bilateral	N/A	N/A	N/A	N/A	N/A
Transverse temporal gyrus	bilateral	N/A	bilateral	N/A	bilateral	N/A
Planum temporale	bilateral	N/A	bilateral	N/A	bilateral	N/A
Planum polare	bilateral	N/A	bilateral	N/A	bilateral	N/A
Parahippocampal gyrus	bilateral	N/A	bilateral	N/A	left	N/A
Precuneus	bilateral	N/A	bilateral	N/A	bilateral	N/A
Supramarginal gyrus	bilateral	N/A	left	N/A	left	N/A
Angular gyrus	bilateral	N/A	left	N/A	left	N/A
Lateral occipital cortex	bilateral	N/A	left	N/A	left	N/A
Hippocampus	bilateral	N/A	bilateral	N/A	left	N/A
Amygdala	bilateral	N/A	bilateral	N/A	left	N/A
Accumbens	bilateral	N/A	bilateral	N/A	bilateral	N/A
Caudate	bilateral	N/A	bilateral	N/A	N/A	N/A
Putamen	bilateral	N/A	bilateral	N/A	N/A	N/A
Thalamus	bilateral	N/A	bilateral	bilateral	bilateral	bilateral
Cuneus	right	N/A	N/A	N/A	N/A	N/A
Occipital pole	left	N/A	N/A	N/A	N/A	N/A
Periaqueductal GM	yes	N/A	yes	yes	yes	yes
Cerebellum	left	N/A	N/A	N/A	N/A	N/A
Peak FWE + Bonferroni-corrected p value	0.002	0.009	0.008	0.009	0.009	0.01
Total volume of clusters with significantly lower GM volume [mm^3^]	82,114.	4,112.	24,446.	6,194.	13,056.	4,770.
Number of GM areas with atrophy	33	4	24	7	21	7
Number of bilateral/unilateral/midsagittal GM areas with atrophy	29/3/1	4/0/0	20/3/1	5/1/1	14/6/1	5/1/1

Abbreviations: GM = grey matter, SF = sustained fatigue, RF = reversible fatigue, NF = never fatigued, EDSS = Expanded Disability Status Scale, CESD = Center for Epidemiological Studies - Depression Scale, FWE = family-wise error.

When controlling for CES-D (in addition to age, sex, disease duration, and EDSS), SF versus NF patients showed significant atrophy in 23 GM areas (19 bilateral), whereas RF versus NF patients showed significant atrophy in 7 GM areas (5 bilateral) (Table 2). The total number of significantly different GM voxels in the SF versus NF contrast was 4-times larger compared to the RF versus NF contrast (Table 2). Of note, one patient’s CES-D was not measured at the time of the MFIS assessment. Instead, we used a CES-D score obtained 34 months earlier.

When controlling for medication (in addition to age, sex, disease duration, EDSS and CES-D), SF versus NF patients showed significant atrophy in 21 GM areas (14 bilateral), whereas RF versus NF patients showed significant atrophy in 7 GM areas (5 bilateral) (Table 2). The total number of significantly different GM voxels in the SF versus NF contrast was nearly 3-times larger compared to the RF versus NF contrast (Table 2).

Bonferroni correction resulted in a 35% reduction in the total number of significantly different GM voxels in the SF versus NF contrast, as well as in an 89% reduction in the RF versus NF contrast when controlling for age, sex disease duration and EDSS. A prominent negative confounding effect of depression was observed bilaterally in the cerebellar cortex in the SF versus NF and in the RF versus NF contrasts, which did not survive Bonferroni correction (Supplementary Figs 1 and 2). This is of note, given the extent and anatomical coherence of the result (Supplementary Figs 1 and 2).

## Discussion

A neurogenic component of MS-related fatigue has been supported by 10 out of 13 previous studies using unbiased image analyses, such as voxel-, tensor-, or automated segmentation-based techniques^4–13^. Overall, our VBM results support an association between neurodegeneration and fatigue in MS patients.

Previous studies categorized patients into “fatigued” or “non-fatigued” groups based on a single time-point fatigue assessment, and their results showed heterogeneity in regional atrophy patterns potentially relevant to fatigue^4–13^.

Our study design used multiple longitudinal assessments of fatigue to improve robustness of group assignment. Stratification of patients according to historical fatigue scores may inform on the different mechanisms involved in the pathophysiology of fatigue. Since inflammatory cytokines, hormones or metabolic factors are likely to induce RF, whereas neurodegeneration may cause SF, we expected that SF patients would show more pronounced GM damage than RF and NF patients.

Our results showed that both SF and RF were associated with neurodegeneration in all GM regions known to be associated with fatigue from previous studies, independently from age, sex, disease duration, EDSS, CES-D and medication. Compared to NF patients, the total number of significantly different GM voxels was more than twenty-times larger in SF than in RF patients, but direct comparison of SF with RF patients showed no significant voxel-wise differences. WM changes (measured by brain WMLL) were significantly more pronounced in SF compared to RF and NF patients, and there was a trend for higher WMLL in RF versus NF patients. These findings suggest that the same neuronal circuitries are affected in RF as in SF patients, albeit to a lesser extent in the former.

Several previous structural MRI studies of fatigue investigated the association of fatigue with WM lesions in MS. However, only a few of these studies found significant association between fatigue and total brain WMLL^4,20,21^, or regional brain WMLL in frontal^8,22^, temporal^8^, parietal, internal capsular and periventricular areas^20^, while other studies using a similar approach failed to do so^16–18^. Our results support the notion that both GM and WM damage play a role in the development of fatigue in MS.

Chaudhuri and Behan associated “central fatigue” with the failure of the non-motor function of the cortico-striato-thalamic loop^23^. This hypothesis has been supported by several neuroimaging studies in MS^1,3^. Other networks, including temporal, parietal and occipital connections may also play a role in the development of fatigue, according to diffusion tensor MRI studies^9,24^ and other MRI studies investigating the localization of MS lesions^4^. We robustly replicated the anatomical patterns of GM atrophy described in previous work. Our findings support the hypothesis that all of the above-mentioned networks, including limbic (frontal-orbital and cingulate cortices), primary sensory-motor (pre- and postcentral gyri), associative (frontal, temporal, parietal, insular and occipital) cortical and subcortical regions (striatum, thalamus, amygdala) might have a role in the development of fatigue in MS. In addition, our study is the first to report association between fatigue and hippocampal atrophy in MS. The prefrontal cortex-hippocampus circuit plays a role not only in memory, attention and decision-making^25^, which are major components of cognitive fatigue, but is involved also in reward mechanisms^25,26^ providing support for the effort-reward imbalance theory of fatigue^3^.

Fatigue and depression scores were highly correlated in our cohort, consistent with previous findings^1,27^. It is worthwhile noting that all subscale scores of the CES-D were higher in SF compared to the RF and NF groups. Therefore, we don’t ascribe the correlation between CES-D and MFIS to the overlap in presence of questions related to somatic symptoms in both CES-D and MFIS questionnaires. Our results support that depression is a significant co-morbidity in fatigued patients and may suggest that fatigue and depression might indeed be mediated by damage to shared pathways. The above-mentioned studies assessed depression using various questionnaires. Nine studies excluded patients based on high depression scores^4,6–9,15,28^ or concomitant therapy with anti-depressants^13^ or history of psychiatric disorders^29^. One study compared patients with only fatigue, only depression and both^10^ and one made no correction for depression^12^. The potential confounding effect of depression in the context of the association between brain damage and fatigue was investigated by 2 previous studies. One showed significant association of fatigue with caudate and accumbens atrophy when controlling for depression and EDSS^11^, while the other study found no significant GM atrophy related to fatigue when accounting for depression^14^. Our voxel-based MRI analyses showed that depression has a significant confounding effect in several frontal, temporal, parietal, occipital and deep GM areas. In the SF versus NF contrast, significant positive confounding effect of depression was observed in the bilateral superior, middle, inferior frontal gyri, the pre- and postcentral gyri, accumbens, and right lateral occipital cortex, supramarginal and angular gyri (Table 2 and Fig. 1). In the RF versus NF contrast, negative confounding effect of depression was observed in the thalamus (Table 2 and Fig. 2). Our results suggest that damage to these areas may play a role in the co-morbid development of fatigue and depression in MS patients. We noted a negative confounding effect on cerebellar cortex. This finding did not survive Bonferroni correction, but, given the extent of the resulting cluster of voxels and its striking anatomical coherence outlining a large part of the cerebellar cortex, further attention is warranted in future studies.

The presence/absence of anti-fatigue, anti-depressant and/or anxiolytic treatments also showed a significant positive confounding effect in SF, and to a lesser extent, in RF patients. The most prominent positive confounding effect was observed in the caudate and putamen in the SF versus NF contrast. Our results suggest that pharmacological treatment is a significant confounder of MS-related fatigue. This observation may pave the way for future studies which aim to investigate the association of global or local (ie, GM region or WM tract-specific) brain damage with anti-fatigue treatment response in MS.

It has been hypothesized that lateralization may exist in fatigued MS patients^30^ based on the findings of Riccitelli *et al*.^7^ who demonstrated correlation between fatigue and atrophy in left precentral gyrus and central sulcus. However, most of our findings were bilateral, including the pre- and postcentral gyri (Table 2). Several previous studies showed both bilateral and unilateral findings^4–13^, but the unilateral ones were not consistently reproduced providing no clear evidence regarding the lateralization of fatigue in MS. In fact, the observed imbalance in the extent and distribution of GM atrophy between SF and RF patients (i.e., 29 out of 33 GM areas showed bilateral spatial pattern in SF, while in RF all 4 GM areas were involved bilaterally) (Table 1).

In previous studies, both RF and NF patients would have been stratified as “non-fatigued’ MS patients. Our results suggest that previous existence of clinically significant fatigue in currently “non-fatigued” patients is associated with GM atrophy, potentially explaining inconsistent findings of previous studies that stratified MS patients using a single fatigue assessment.

Our groups were selected from the CLIMB cohort based on longitudinal MFIS scores and matched based on age, gender, disease duration and EDSS. Due to this selection and matching process, the pooled SF + RF + NF cohort showed significantly higher disease duration, female-to-male ratio and cognitive fatigue compared to the CLIMB cohort.

Our study has limitations, including: (1) time between MRI and fatigue assessment varied across participants, with MRI scans within a month of MFIS assessment in only 57 out of 98 patients. (2) In our statistical analyses, correction was made only for age, sex, disease duration, EDSS and depression, but not for other potential confounders of fatigue, such as anxiety, physical activity and sleep problems. (3) Treatment with anti-fatigue, anxiolytics, anti-depressant, disease-modifying drugs, monthly iv steroids and/or immunosuppressants were not exclusion criteria. (4) While this may be the first time that patients were classified according to fatigue patterns derived from repeated measures, the frequency of these measures was constrained by the retrospective nature of this work, and requires further consideration. (4) We used the Bonferroni method to correct for multiple comparisons (in addition to FEW correction), which is applicable when the number of tests is less than 5. However, this method is not as powerful as the Tukey method^31^.

Future prospective studies of MS-related fatigue should take into consideration temporal patterns of fatigue. However, the most adequate frequency of fatigue assessments for optimal, pathologically relevant patient stratification remains to be determined. Novel approaches to fatigue assessment, including the use of mobile technologies for frequent, real-time assessments, are likely to lead to better understanding of the pathophysiology of fatigue and other interrelated symptoms.

## Materials and Methods

### Participants

MS patients were selected from the Quality Of Life (QOL) subset of our longitudinal cohort study of over 2000 MS patients, named Comprehensive Longitudinal Investigations of MS at the Brigham and Women’s Hospital (CLIMB) (http://partnersmscenter.org/clinical-programs/climb-study/) (Table 1). The QOL subset (n > 800) undergoes annual MRI and neurological examination and biennial QOL assessments, including fatigue and depression measurements, using the Modified Fatigue Impact Scale (MFIS)^32,33^ and the Center for Epidemiological Studies Depression Scale (CES-D)^34^, respectively. The MFIS has three domains (i.e., cognitive, physical and psychosocial) and its cut-off for clinically relevant fatigue is 38 (total score including all domains)^33^, whereas CES-D has four domains (i.e, somatic symptoms, depressed affect, amhedonia, interpersonal concerns)^35^ and scores ≥16 (including all domains) are in the depressed range^36^. The QOL assessments were performed on the day of the neurological visit.

#### Definition of fatigue subgroups

In the current study, the following patient groups were defined based on retrospective longitudinal MFIS scores: (i) SF: last two consecutive MFIS ≥38, (ii) RF: most recent MFIS <38 and at least one prior MFIS ≥38; (iii) NF: no MFIS ≥38 (minumum 5 assessments needed). We queried the CLIMB database on 02/19/16 and found 123 SF, 98 RF and 238 NF patients out of the QOL subgroup of 859 MS patients. Patients without 3T MRI were excluded and the closest 3T MRI scan to the latest MFIS measurement was selected for image analysis in the remaining patients. Further restriction criteria were applied: no clinically isolated syndrome; no history of psychotic disorder, major neurologic disorder (other than MS) or malignancies; EDSS ≤6; no clinical relapse/acute intravenous streroid treatment within 90 days before the MFIS assessment or the MRI scan or between the MFIS and MRI assessments. To maximize the number of SF patients, the maximum difference in time between the last MFIS assessment and MRI scan was set at 15 months and the upper limit of age at 66 years. Then, we matched the SF group with the other 2 groups based on age, sex, disease duration and EDSS. We identified 30 SF, 31 RF and 37 NF patients (Table 1). Several patients were treated with anti-fatigue medications (modafinil, armodafinil, amphetamine, amantadine or methylphenidate) (47% of SF, 23% of RF, 30% of NF patients); anxiolytics (27% of SF, 16% of RF, 8% of NF patients); anti-depressants (47% of SF, 26% of RF, 22% of NF) and 67% of SF, 68% of RF and 57% of NF patients received at least one of these drugs. Most of the patients were on disease-modifying treatment (87% of SF, 80% of RF, 92% of NF patients), 2 SF and 2 RF patients received monthly intravenous steroids, and 1 SF and 1 NF patients were on immunosuppressant (mycophenolate mofetil) treatment. This study was approved by the Institutional Review Board of our Institution (Partners Healthcare) and all research was performed in accordance with relevant guidelines and regulations. The CLIMB Study group obtained informed consent from all patients whose clinical and MRI data were analyzed in this study.

### Magnetic resonance imaging

Brain images were acquired using a 3 Tesla Siemens Skyra scanner as follows: (1) Sagittal 3D T1-weghted MPRAGE: TR/TE/TI = 2300/2.96/900 ms, voxel sixe = 1 × 1 × 1 mm^3^, FOV = 256 mm, flip angle = 9 deg, matrix size = 256 × 240 and (2) Sagittal 3D T2-weighted FLAIR: TR/TE/TI = 5000/389/1800 ms, voxel size = 1 × 1 × 1 mm^3^, FOV = 256 mm, flip angle = 120 deg, matrix size = 256 × 240.

### Lesion segmentation

White matter (WM) lesions were segmented using the lesion growth algorithm module of the lesion segmentation toolbox (LST v2.0.15) in Statistical Parametric Mapping (SPM)12^37^. This algorithm first segments the T1-weighted images into three main tissue classes (GM, WM, cerebrospinal fluid (CSF)), then this information is combined with the coregistered FLAIR intensities to calculate lesion belief maps. Based on visual evaluation, a kappa value of 0.1 was selected as optimal threshold for the computation of binary lesion maps. The automatically-generated lesion maps were inspected and manually edited in 3D-Slicer (https://www.slicer.org) to erase false positives only in GM areas. Total brain WM lesion load (WMLL) was calculated using 3D-Slicer. Finally, the lesions were filled on T_1_-weighted images using the lesion filling module of the LST in preparation for voxel-based morphometry (VBM).

### Voxel based morphometry

VBM was performed on T1-weighted images to detect differences in brain GM atrophy between the three groups^38^. Supra- and infratentorial WM, brainstem, and lesions were masked, and excluded from analysis. T_1_-weighted images were preprocessed using the VBM toolbox in SPM12^39^. We used diffeomorphic anatomical registration through exponentiated lie algebra (DARTEL)^40^ to create a study-specific template and register the images to the ICBM 152 template (MNI space). The images were then merged using *fslmerge*, and smoothed with a Gaussian kernel (σ = 4 mm) using *fslmaths* (https://fsl.fmrib.ox.ac.uk/fsl/fslwiki/Fslutils).

### Cerebellum segmentation

T1-weighted images were processed with CERES^41^, an automated atlas-based cerebellum segmentation tool to calculate total cerebellum volume, cerebellar GM volume and cerebellar cortical thickness.

### Statistical analysis

Comparison of continuous clinical variables (1) between SF, RF and NF groups was performed using one-way ANOVA and (2) between the pooled SF + RF + NF cohort and the CLIMB study cohort was performed using Wilcoxon rank sum test. Differences in male/female ratio were assessed by Pearson’s chi-square test. We assessed associations among MFIS, CES-D and EDSS scores using Spearman’s rank correlation. WMLL was compared between the groups using one-way ANOVA.

For voxel-based analysis, we used nonparametric permutations (n = 5000) implemented in FSL-Randomise (https://fsl.fmribox.ac.uk/fsl/fslwiki/Randomise). Threshold-free cluster enhancement was used to adjust for family-wise error (FWE) correction for multiple comparisons^42,43^. In addition, Bonferroni correction was used to correct for the number of pairwise comparisons (ie, SF versus NF, SF versus RF, RF versus NF). Accordingly, voxels with a FWE + Bonferroni-corrected *p* < 0.017 were considered significant.

To investigate the effect of depression on the relationship between fatigue and GM atrophy, we performed secondary analysis controlling for CES-D (in addition to age, sex, disease duration and EDSS). We also assessed the voxel-wise association between depression severity (continuous CES-D score) and brain GM atrophy in the pooled patient cohort, controlling for age, sex, disease duration and EDSS. To account for the effects of medications that may lower fatigue and/or depression levels, in a separate model, we added medication, as a dichotomous variable: 1 = received anti-fatigue and/or anti-depressant and/or anxiolytic treatment, 0 = received none of these medications. Stata13 (StataCorp, College Station, Texas, USA) was used for all statistical analyses except for VBM.

## Supplementary information

Supplementary Figures

## Data Availability

The datasets generated during and/or analyzed during the current study are available from the corresponding author on reasonable request.

## References

[1] Induruwa I, Constantinescu CS, Gran B (2012). Fatigue in multiple sclerosis - a brief review. J Neurol Sci.

[2] Cavallari M (2016). Fatigue predicts disease worsening in relapsing-remitting multiple sclerosis patients. Mult Scler.

[3] Dobryakova E, DeLuca J, Genova HM, Wylie GR (2013). Neural correlates of cognitive fatigue: cortico-striatal circuitry and effort-reward imbalance. J Int Neuropsychol Soc.

[4] Sepulcre J (2009). Fatigue in multiple sclerosis is associated with the disruption of frontal and parietal pathways. Mult Scler.

[5] Andreasen AK (2010). Regional brain atrophy in primary fatigued patients with multiple sclerosis. Neuroimage.

[6] Calabrese M (2010). Basal ganglia and frontal/parietal cortical atrophy is associated with fatigue in relapsing-remitting multiple sclerosis. Mult Scler.

[7] Riccitelli G (2011). Voxelwise assessment of the regional distribution of damage in the brains of patients with multiple sclerosis and fatigue. AJNR Am J Neuroradiol.

[8] Derache N (2013). Fatigue is associated with metabolic and density alterations of cortical and deep gray matter in Relapsing-Remitting-Multiple Sclerosis patients at the earlier stage of the disease: A PET/MR study. Mult Scler Relat Disord.

[9] Rocca MA (2014). Regional but not global brain damage contributes to fatigue in multiple sclerosis. Radiology.

[10] Hanken K, Eling P, Klein J, Klaene E, Hildebrandt H (2016). Different cortical underpinnings for fatigue and depression in MS?. Mult Scler Relat Disord.

[11] Damasceno A, Damasceno BP, Cendes F (2016). Atrophy of reward-related striatal structures in fatigued MS patients is independent of physical disability. Mult Scler.

[12] Nourbakhsh B (2016). Longitudinal associations between brain structural changes and fatigue in early MS. Mult Scler Relat Disord.

[13] Cruz Gomez AJ, Ventura Campos N, Belenguer A, Avila C, Forn C (2013). Regional brain atrophy and functional connectivity changes related to fatigue in multiple sclerosis. PLoS One.

[14] Gobbi C (2014). Influence of the topography of brain damage on depression and fatigue in patients with multiple sclerosis. Mult Scler.

[15] Finke C (2015). Altered basal ganglia functional connectivity in multiple sclerosis patients with fatigue. Mult Scler.

[16] van der Werf SP (1998). Fatigue in multiple sclerosis: interrelations between fatigue complaints, cerebral MRI abnormalities and neurological disability. J Neurol Sci.

[17] Palotai Miklos, Mike Andrea, Cavallari Michele, Strammer Erzsebet, Orsi Gergely, Healy Brian C, Schregel Katharina, Illes Zsolt, Guttmann Charles RG (2017). Changes to the septo-fornical area might play a role in the pathogenesis of anxiety in multiple sclerosis. Multiple Sclerosis Journal.

[18] Bakshi R (1999). Fatigue in multiple sclerosis: cross-sectional correlation with brain MRI findings in 71 patients. Neurology.

[19] Johansson S, Ytterberg C, Hillert J, Widen Holmqvist L, von Koch L (2008). A longitudinal study of variations in and predictors of fatigue in multiple sclerosis. J Neurol Neurosurg Psychiatry.

[20] Colombo B (2000). MRI and motor evoked potential findings in nondisabled multiple sclerosis patients with and without symptoms of fatigue. J Neurol.

[21] Tedeschi G (2007). Correlation between fatigue and brain atrophy and lesion load in multiple sclerosis patients independent of disability. J Neurol Sci.

[22] Morgante F (2011). Is central fatigue in multiple sclerosis a disorder of movement preparation?. J Neurol.

[23] Chaudhuri A, Behan PO (2000). Fatigue and basal ganglia. J Neurol Sci.

[24] Gobbi C, Rocca MA, Pagani E, Riccitelli GC, Pravatà E, Radaelli M, Martinelli-Boneschi F, Falini A, Copetti M, Comi G, Filippi M (2014). Forceps minor damage and co-occurrence of depression and fatigue in multiple sclerosis. Multiple Sclerosis Journal.

[25] Wall PM, Messier C (2001). The hippocampal formation–orbitomedial prefrontal cortex circuit in the attentional control of active memory. Behavioural brain research.

[26] Ito R, Lee AC (2016). The role of the hippocampus in approach-avoidance conflict decision-making: Evidence from rodent and human studies. Behavioural brain research.

[27] Wood B (2013). Prevalence and concurrence of anxiety, depression and fatigue over time in multiple sclerosis. Mult Scler.

[28] Andreasen AK, Spliid PE, Andersen H, Jakobsen J (2010). Fatigue and processing speed are related in multiple sclerosis. Eur J Neurol.

[29] Genova HM (2013). Examination of cognitive fatigue in multiple sclerosis using functional magnetic resonance imaging and diffusion tensor imaging. PLoS One.

[30] Chen Q (2011). Neuroimaging by voxel-based morphometry: possible approach to finding the correlation between brain structural changes and fatigue severity in patients with multiple sclerosis. AJNR. American journal of neuroradiology.

[31] Bender R, Lange S (2001). Adjusting for multiple testing–when and how?. J Clin Epidemiol.

[32] Guidelines, M. S. C. f. C. P. *Fatigue and multiple sclerosis: evidence-based management strategies for fatigue in multiple sclerosis*. (Multiple Sclerosis Council for Clinical Practice Guidelines, 1998).

[33] Amtmann D (2012). Comparison of the psychometric properties of two fatigue scales in multiple sclerosis. Rehabilitation psychology.

[34] Radloff LS (1977). The CES-D scale a self-report depression scale for research in the general population. Applied psychological measurement.

[35] Radloff LS (1991). The use of the Center for Epidemiologic Studies Depression Scale in adolescents and young adults. J Youth Adolesc.

[36] Lewinsohn PM, Seeley JR, Roberts RE, Allen NB (1997). Center for Epidemiologic Studies Depression Scale (CES-D) as a screening instrument for depression among community-residing older adults. Psychology and aging.

[37] Schmidt P (2012). An automated tool for detection of FLAIR-hyperintense white-matter lesions in Multiple Sclerosis. Neuroimage.

[38] Ashburner J, Friston KJ (2000). Voxel-based morphometry–the methods. Neuroimage.

[39] Ashburner J (2012). SPM: a history. Neuroimage.

[40] Ashburner J (2007). A fast diffeomorphic image registration algorithm. Neuroimage.

[41] Romero JE (2017). CERES: A new cerebellum lobule segmentation method. Neuroimage.

[42] Smith SM, Nichols TE (2009). Threshold-free cluster enhancement: addressing problems of smoothing, threshold dependence and localisation in cluster inference. Neuroimage.

[43] Nichols TE, Holmes AP (2002). Nonparametric permutation tests for functional neuroimaging: a primer with examples. Hum. Brain Mapp..

