# Supplementary material for: History of fatigue in multiple sclerosis is associated with grey matter atrophy
Source: Sci Rep. 2019 Oct 14;9:14781. doi: 10.1038/s41598-019-51110-2 (PMC6791855; doi:10.1038/s41598-019-51110-2)
Supplement: Supplementary file 1 — Supplementary Figures [file 41598_2019_51110_MOESM1_ESM.pdf]

Full title:

History of fatigue in multiple sclerosis is associated with grey matter atrophy.

Authors:

*Miklos Palotai MD[1], *Aria Nazeri MD[2], Michele Cavallari MD, PhD[1], Brian C. Healy PhD[3,4], Bonnie Glanz PhD[3], Stefan M. Gold PhD[5], Howard L. Weiner MD[3], Tanuja Chitnis MD[3], Charles R.G. Guttmann MD[1]

Affiliations:

[1] Center for Neurological Imaging, Department of Radiology, Brigham and Women's Hospital, Harvard Medical School, Boston, Massachusetts, USA

[2] Mallinckrodt Institute of Radiology, Washington University School of Medicine, St. Louis, Missouri, USA

[3] Partners Multiple Sclerosis Center, Department of Neurology, Brigham and Women's Hospital, Harvard Medical School, Boston, MA, USA

[4] Biostatistics Center, Massachusetts General Hospital, Harvard Medical School, Boston, Massachusetts, USA

[5] Charité Universitätsmedizin Berlin, Klinik für Psychiatrie und Medizinische Klinik m.S. Psychosomatik, Berlin, Germany; Institut für Neuroimmunologie und Multiple Sklerose (INIMS), Universitätsklinikum Hamburg-Eppendorf, Hamburg, Germany

*These authors contributed equally to the manuscript.

Corresponding author:

Charles R.G. Guttmann, M.D.

Director, Center for Neurological Imaging

Brigham and Women`s Hospital, Harvard Medical School

1249 Boylston street, Boston, MA 02215

e-mail: guttmann@bwh.harvard.edu

Tel: +1 (617) 278-0613

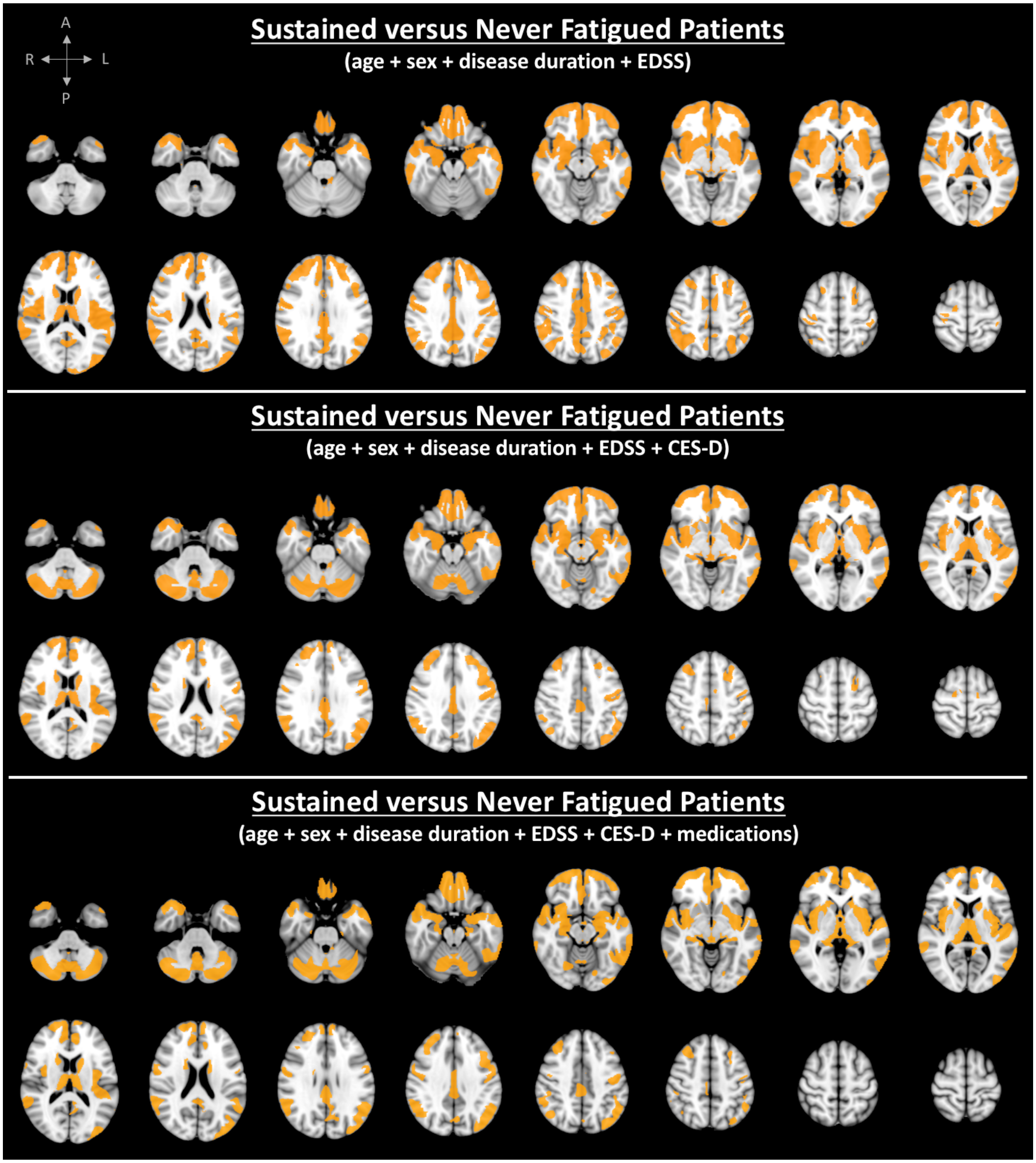

Supplementary Figure 1: Spatial distribution of clusters with significant atrophy overlaid on the ICBM 152 template in MS patients with sustained fatigue (SF) compared to never fatigued (NF) MS patients. Correction was made for age, sex, disease duration and Expanded Disability Status Scale (EDSS) score (top), and for Center for Epidemiological Studies - Depression score (CES-D) (middle), as well as for medication (bottom). (orange labels = family-wise error-corrected p value<0.05).

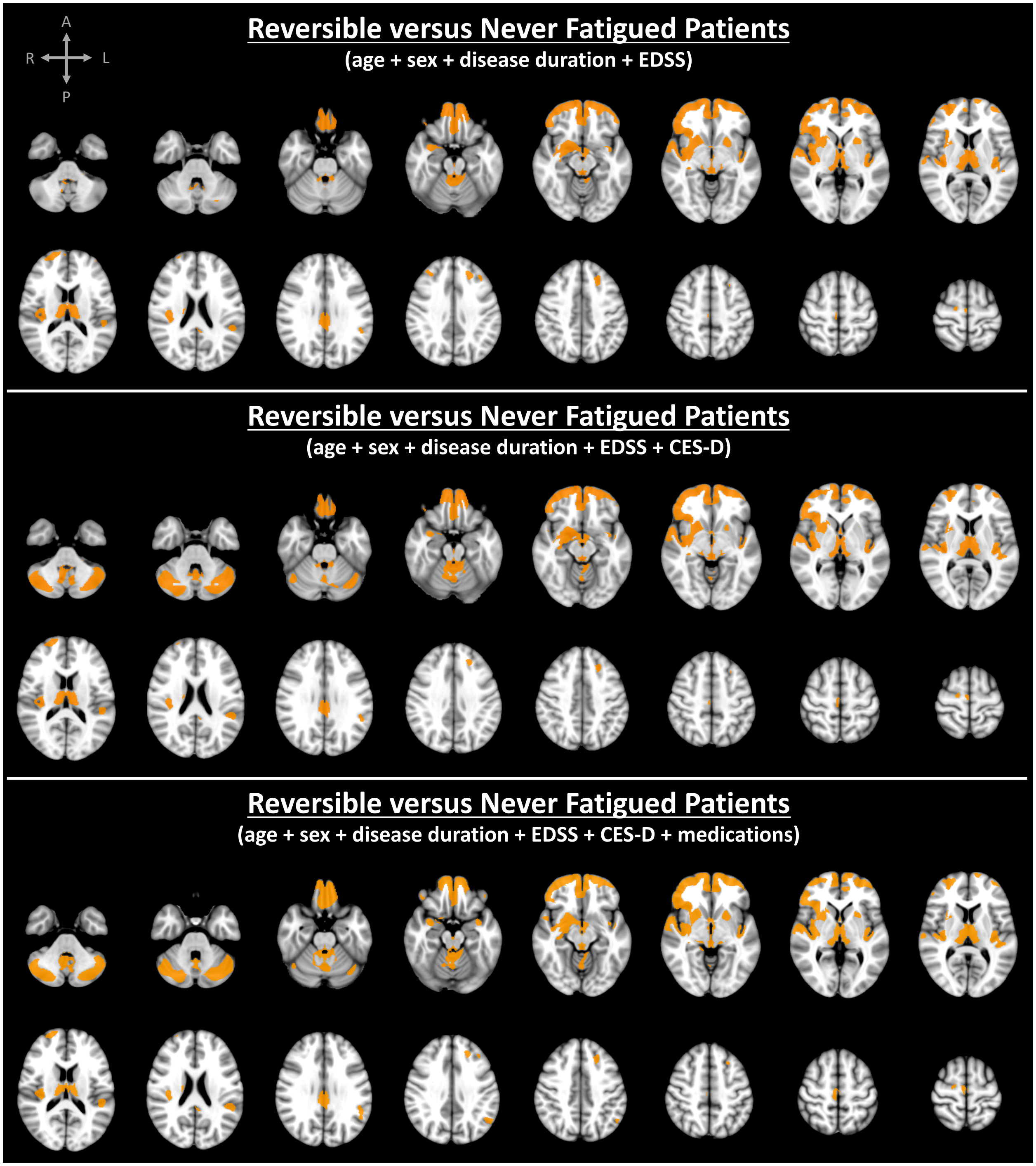

Supplementary Figure 2: Spatial distribution of clusters with significant atrophy overlaid on the ICBM 152 template in MS patients with reversible fatigue (RF) compared to never fatigued (NF) MS patients. Correction was made for age, sex, disease duration and Expanded Disability Status Scale (EDSS) score (top), and for Center for Epidemiological Studies - Depression score (CES-D) (bottom), as well as for medication (bottom) (orange labels = family-wise error-corrected p value<0.05).
